# mTORC1 signaling pathway regulates tooth repair

**DOI:** 10.1038/s41368-023-00218-3

**Published:** 2023-03-16

**Authors:** Honghong Liu, Yu Yue, Zhiyun Xu, Li Guo, Chuan Wu, Da Zhang, Lingfei Luo, Wenming Huang, Hong Chen, Deqin Yang

**Affiliations:** 1 Department of Endodontics, Stomatological Hospital of Chongqing Medical University, Chongqing, China; 2 Stomatological Hospital of Chongqing Medical University, Chongqing, China; 3 Chongqing Key Laboratory of Oral Diseases and Biomedical Sciences, Chongqing, China; 4 Chongqing Municipal Key Laboratory of Oral Biomedical Engineering of Higher Education, Chongqing, China; 5 Institute of Developmental Biology and Regenerative Medicine, Southwest University, Chongqing, China

**Keywords:** Genetics, Developmental biology

## Abstract

Tooth germ injury can lead to abnormal tooth development and even tooth loss, affecting various aspects of the stomatognathic system including form, function, and appearance. However, the research about tooth germ injury model on cellular and molecule mechanism of tooth germ repair is still very limited. Therefore, it is of great importance for the prevention and treatment of tooth germ injury to study the important mechanism of tooth germ repair by a tooth germ injury model. Here, we constructed a *Tg(dlx2b:Dendra2-NTR)* transgenic line that labeled tooth germ specifically. Taking advantage of the NTR/Mtz system, the *dlx2b*^+^ tooth germ cells were depleted by Mtz effectively. The process of tooth germ repair was evaluated by antibody staining, in situ hybridization, EdU staining and alizarin red staining. The severely injured tooth germ was repaired in several days after Mtz treatment was stopped. In the early stage of tooth germ repair, the expression of phosphorylated 4E-BP1 was increased, indicating that mTORC1 is activated. Inhibition of mTORC1 signaling in vitro or knockdown of mTORC1 signaling in vivo could inhibit the repair of injured tooth germ. Normally, mouse incisors were repaired after damage, but inhibition/promotion of mTORC1 signaling inhibited/promoted this repair progress. Overall, we are the first to construct a stable and repeatable repair model of severe tooth germ injury, and our results reveal that mTORC1 signaling plays a crucial role during tooth germ repair, providing a potential target for clinical treatment of tooth germ injury.

## Introduction

Local thickening of oral epithelium is the initial morphological sign of tooth development; subsequently, thickened epithelium together with underlying mesenchyme produce the tooth germs, which will develop into a functional tooth.^1^ The tooth germ development is a long-term and complex process. Once impaired, the tooth germ can lead to tooth hard tissues hypoplasia, tooth morphological alteration, tooth loss, cyst formation, and even dental-facial deformities.^2–4^ Furthermore, the initial steps of digestion and absorption occur in the mouth, so conditions mentioned above will lead to decreased chewing efficiency, which can significantly affect the intake of nutrients and thus threaten people’s health.^5^ Traditional treatment methods such as fixed prosthetic crowns and implant dentures can only partially restore facial beauty and function. However, these repair methods still cannot replace natural teeth for lack of biological activity. Therefore, understanding the repair mechanism of the injured tooth germ deeply and inducing tooth germ repair has attracted great attention.

Mammals such as mice, beagles, and miniature pigs were always used as experimental animal models in previous studies on tooth development and regeneration.^6–8^ However, these animal models have several limitations. First, mammalian embryonic development occurs in the uterus, and there are severe limitations on observation, intervention, and dynamic real-time tracking of early tooth development. Nextly, mammalian tissue regeneration is degraded, while mice do not even have tooth replacement.^9^ Therefore, it is necessary to find more effective model animals to construct the novel tooth germ injury model. Zebrafish, as a new experimental model animal, has a strong ability to regenerate multiple tissues and organs.^10–12^ Zebrafish also has the advantages of in vitro fertilization and transparent embryos.^13,14^ Fluorescently labeled zebrafish strains can be visualized by transgenic methods to explore the complex mechanisms involved in development, repair and regeneration.^15^ Most importantly, the pharyngeal teeth of zebrafish undergo bud stage, cap stage and early bell stage, which are very similar to the development process of human tooth germ.^16^ These obvious advantages make zebrafish a suitable animal model to observe tooth development-related conditions.^17–19^ Previous researchers used zebrafish as model animals to study tooth development and replacement.^20–22^ However, the research of tooth germ injury in zebrafish pharynx is very rare, and there are even no studies on the tooth germ repair in zebrafish.

The mammalian target of rapamycin complex 1 (mTORC1) signaling is highly conserved in eukaryotes.^23^ mTORC1 is mainly composed of rapamycin-sensitive aptamer protein Raptor, which is critical for mTORC1 activity.^24–26^ mTORC1 is primarily regulated by the phosphorylation of p70S6 kinase 1 (S6K) and eIF4E-binding protein (4E-BP). Studies have shown that inhibition of mTORC1 can disrupt tooth development, leading to defective tooth formation and malformations.^27–29^ Moreover, the signaling pathways of development and regeneration are closely related. However, to our knowledge, whether mTORC1 signal regulates the repair of severe tooth germ injury has not been reported, which is definitely worth further investigations. The incisors of mice wear away constantly but continue to grow. This lifelong growth is driven by both dental epithelial cells and dental mesenchymal stem cells located in the growth center of the incisor,^30^ which allows continuous repair of the incisor after damage. In this study, in order to further verify the conservation of mTORC1 signaling pathway in mammals, we injected 2-month-old mice with rapamycin or 3-month-old mice with L-Leucine to observe the effect of inhibiting or promoting mTORC1 signaling pathway on incisors restoration.

The nitroreductase (NTR)/metronidazole (Mtz) system^31^ can induce target cell specific expression of NTR by the transgene. NTR can bind to Mtz after being reduced, transforming non-toxic Mtz into cytotoxic metabolites and ultimately resulting in the death of cells expressing NTR specifically. The destructiveness of this method does not spread to neighboring cells, causing non-specific cell death. Therefore, this injury method is more straightforward, consistent, and repeatable than surgery or laser injury.^32^ The key to adopt this injury system is to determine the specific expressed genes of target cells. In zebrafish, *dlx2b* is expressed in the mesenchymal and epithelial tissues of the tooth germ during early tooth development but not expressed in alveolar bone or other surrounding tissues. However, it is not expressed alveolar bone or other surrounding tissues. Therefore, *dlx2b* is a specific marker gene of the zebrafish tooth germ.^33,34^ In this study, we first constructed the *Tg(dlx2b:Dendra2-NTR)* transgenic line, and then NTR/Mtz system was used to specifically induce tooth germs injury at a specific time, after that, the restoration of zebrafish tooth germ was observed. Subsequently, we screened and explored the activated signal pathway after injury. We demonstrated that mTORC1 was activated in the early stage and investigated whether mTORC1 was involved in regulating the repair of the injured tooth germ.

## Results

### Construction of the severe tooth germ injury model based on transgenic line *Tg(dlx2b:Dendra2-NTR*)

**Fig. 1:**
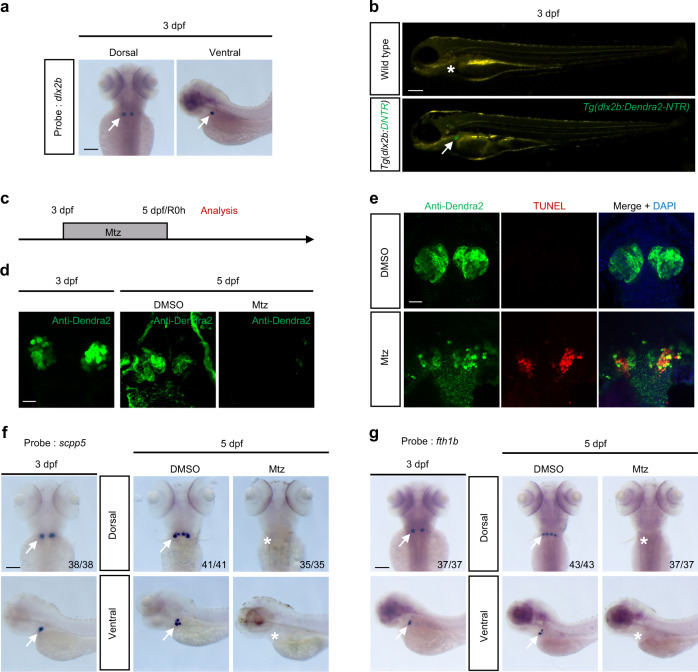
Construction of the severe tooth germ injury model based on transgenic line *Tg(dlx2b:Dendra2-NTR)*. **a** Expression pattern of *dlx2b* at a 3 dpf. Dorsal and ventral views are shown. The probe was *dlx2b*. The white arrows point to the probe-binding site. **b** Total body images of wild-type and transgenic line *Tg(dlx2b:Dendra2-NTR)* at 3 dpf. The white arrows point to the site of fluorescence expression, and the white “*” indicates no obvious fluorescence expression. **c** Embryos at 3 dpf were treated with Mtz for 48 h. **d** Antibody staining of Dendra2 protein (green) representing tooth germ was performed on the 3 dpf and 5 dpf embryos. **e** TUNEL staining was performed after 12 h of Mtz treatment. Apoptosis (red) occurred at the tooth germ (green) location. **f**, **g** In situ hybridization was performed at 3 dpf and 5 dpf intervals. Dorsal and ventral views are presented. The probes were *scpp5* and *fth1b*, respectively. The white arrows point to the probe-binding site, and the white “*” indicates no obvious probe-binding site. The number in the lower right corner is the number of positive/total number of experiments. Scale bar for **a**, **b**, **f**, and **g** = 100 μm; scale bar for **d** and **e** = 20 μm

The expression pattern of *dlx2b* at 3 days post fertilization (dpf) was analyzed by in situ hybridization. It was observed that *dlx2b* was expressed in the pharyngeal teeth area of zebrafish, but not in the surrounding tissues, allowing easy and clear identification and observation of tooth germs (Fig. 1a). A 4136-bp fragment upstream of the *dlx2b* translation initiation site was cloned from the whole genome of zebrafish and then integrated into a vector containing Dendra2-NTR, which has been previously reported.^35^ The recombinant plasmid was named *dlx2b:Dendra2-NTR* (Fig. S1a). Embryos injected with recombinant plasmid and expressing green fluorescence in tooth germs at 3 dpf were designated as filial generation 0 (F0). Adults of F0 were crossed twice with wild-type fish; the heterozygous F2 with a stable fluorescence expression was selected to maintain the line. After F2 in-cross, homozygous fish lines were characterized and applied for the following experiments. As shown in Fig. 1b, no fluorescence expression was observed in the wild-type fish line, but green fluorescence was showed in the tooth germ of the transgenic line. FISH experiments were performed to verify that the fluorescence-expressing cells in the transgenic line were indeed *dlx2b* gene-expressing cells. The results showed that the green fluorescence (Anti-Dendra2) expression was co-located with the red fluorescence (*dlx2b* gene probe expression), indicating that the transgenic line was stable and effective (Fig. S1b). The fluorescence signal of the tooth germ gradually decreased under stereomicroscopy at 3, 4, and 5 dpf due to the growth of muscles and bones during embryonic development (Fig. S1c). Therefore, the expression of *dlx2b*, which represented the tooth germ, was reflected by the antibody staining of Dendra2 protein (anti-Dendra2) in subsequent experiments.

After the transgenic line was successfully constructed, we tried different treatment concentrations of Mtz and found that the most effective working concentration of Mtz was 12 mM (Fig. S1d). The embryos at 3 dpf were treated with Mtz for 48 h (Fig. 1c). Antibody staining showed that tooth germ cells exhibited strong green fluorescence in the dimethyl sulfoxide (DMSO) group (control group), but the fluorescence disappeared in the Mtz group (Fig. 1d). Apoptosis of tooth germ cells was observed to determine whether Mtz treatment led to cell death or just caused loss of *dlx2b* expression. We found that the TUNEL^+^ signal was increased significantly in the Mtz-treated group (Fig. 1e). In situ hybridization was also used to detect the expressions of other maker genes in tooth germ cells after Mtz treatment. Compared with the DMSO group, the expression of *scpp5*, a common marker gene of odontoblasts and ameloblasts,^36^ decreased significantly in the Mtz treatment group. Similarly, the expression of *fth1b*, a gene associated with the mineralization of pharyngeal teeth,^37^ also disappeared in the Mtz treatment group (Fig. 1f, g). All the above findings demonstrated that the tooth germ cells were almost completely ablated after Mtz treatment, indicating that the zebrafish tooth germ severe injury model based on this transgenic line was constructed successfully.

### *Tg(dlx2b:Dendra2-NTR*) could be repaired effectively after severe injury of the tooth germ

**Fig. 2:**
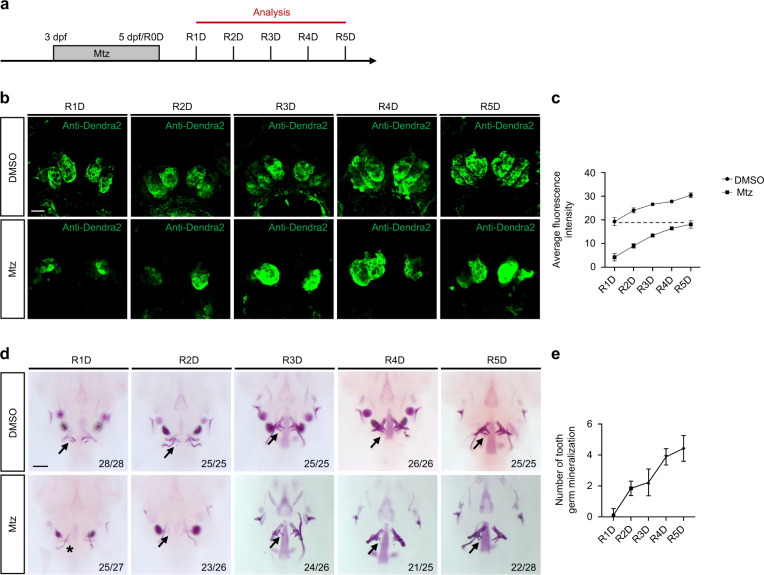
*Tg(dlx2b:Dendra2-NTR)* could be repaired effectively after severe injury of the tooth germ. **a** Embryos from R1D to R5D were analyzed after Mtz treatment. **b** Antibody staining of Dendra2 was performed on R1–5D embryos to show the tooth germ (green) repair process. **c** The average fluorescence intensity of each group (**b**) was analyzed (*n* = 3). **d** Alizarin Red staining was performed on R1–5D embryos. The black arrow points to the mineralized pharyngeal teeth, and the black “*” represents no apparent mineralization. **e** The number of mineralized pharyngeal teeth in each period in the Mtz-treated group was analyzed. The number in the lower right corner is the number of positive/total number of experiments. Scale bar for **d** = 100 μm; scale bar for **b** = 20 μm

The repair process of tooth germs was sequentially observed from day 1 to 5 after Mtz treatment and recorded as R1–5D (repair 1–5 day) (Fig. 2a). The green fluorescence of *dlx2b:Dendra2-NTR*^*+*^ tooth germs recovered, and the intensities got more stronger in the Mtz-treated group from R1D to R5D. At R5D, the average fluorescence intensity in the Mtz-treated group was almost equal to that at R1D in the DMSO group (Fig. 2b, c). The expression of *scpp5* and *fth1b* was detected by in situ hybridization. In the Mtz-treated group, two *scpp5* expression sites were observed in the tooth germ region at the beginning of R1D, and the expression sites increased gradually over time. During R1D-R3D period, most of them still comprised a pair of expression sites. However, it increased to two pairs of expression sites during R4D-R5D period (Fig. S2a, b). The recovery of *fth1b* expression sites during R1D-R5D period was similar to that of *scpp5* (Fig. S2a, d). It can be concluded from the above findings that severely injured tooth germ could be repaired, moreover, the sequence of tooth germ repair was one by one, rather than simultaneous restoration of several pairs of tooth germ. The Alizarin Red staining was performed to further estimate whether the repaired tooth germs could be mineralized into pharyngeal teeth. Alizarin Red can form a complex with calcium salt in the form of chelation to produce a red deposit that can be used to identify mineralized components in tissues and cells.^38^ After Mtz treatment, no evident mineralization deposits were observed in the pharyngeal area at R1D, indicating that mineralization did not occur at the initial stage of tooth germ recovery. The first pair of repaired mineralized pharyngeal teeth began mineralization at R2D and completed mineralization at R3D, the second pair of repaired pharyngeal teeth initiate matrix mineralization at R4D and accomplished mineralization at R5D. To sum up, all of these suggested that the newly repaired tooth germs had the same ability to mineralize into pharyngeal teeth as the uninjured tooth germs (Fig. 2d, e).

### mTORC1 signaling was activated in the early stage of tooth germ repair in zebrafish

**Fig. 3:**
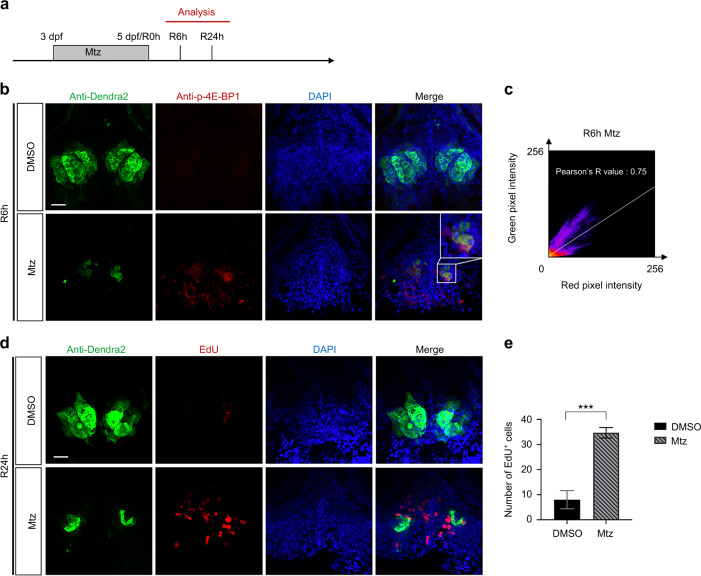
mTORC1 signaling was activated in the early stage of tooth germ repair. **a** Embryos were analyzed at R6h and R24h after Mtz treatment. **b** Antibody staining was performed on R6h embryos, and p-4E-BP1 (red) was located by confocal laser scanning. p-4E-BP1 was co-stained with the tooth germ (green) at R6h. **c** Co-localization analysis of p-4E-BP1 (red) and tooth germ (green) in the Mtz-treated group at R6h. **d**, **e** The cell proliferation (red) was displayed in tooth germ (green) and surrounding anatomical position at R24h by EdU staining, and the statistical data of EdU^+^ cells were analyzed (*n* = 3). Scale bar for **b** and **d** = 20 μm (***P ＜ 0.001)

Tissue repair is inseparable from cell proliferation.^39^ Many signaling pathways function in cell proliferation; therefore, we first used qRT-PCR for preliminary screening. According to qRT-PCR results, mTOR expression was increased significantly after injury (Fig. S3a). We subsequently observed that mTOR expression significantly increased at 0 h of repair (R0h) and R8h, but decreased at R24h after Mtz treatment (Fig. S3b). Since the mTOR signaling pathway mainly functions by mTORC1, we further investigated whether mTORC1 signaling was involved in the tooth germ repair in our model. We examined the expression of phosphorylated-4E-BP1 (p-4E-BP1) and phosphorylated-S6K (p-S6K) at R6h and R24h after Mtz treatment (Fig. 3a). The immunofluorescent staining results indicated that p-4E-BP1 expression (red fluorescence) increased and co-stained with tooth germ cells (green fluorescence) in the Mtz-treated group at R6h (Fig. 3b). However, almost no red fluorescence was detected in the DMSO control group. At R24h, p-4E-BP1 expression decreased in the Mtz-treated group and did not co-stain with tooth germ cells (Fig. S3c). Fiji software was used to analyze the fluorescence co-location images more accurately in the Mtz-treated group at R6h and R24h. Pearson’s *R* value was used to quantify the intensity distribution relationship between the two channels. A value of >0.5 indicates co-location; a value of <0.5 means no co-location. According to Figs. 3c and S3d, Pearson’s *R* value at R6h was 0.75, indicating strong co-location between green and red fluorescence. The value at R24h was 0.11, indicating no apparent co-location. Moreover, p-S6K (red fluorescence) was not significantly detected in control group and Mtz-treated group (Fig. S4a), indicating that p-4E-BP1, rather than p-S6K, was the mainly activated protein during tooth germ repair. In addition, we also detected the cell proliferation of the tooth germ at R24h. In the EdU staining results, compared with the control group, cell proliferation increased significantly in the Mtz-treated group (Fig. 3d, e), suggesting that the cell proliferation of the tooth germ and its surrounding area was activated during the repair process.

### Rapamycin inhibited the repair of severely injured tooth germs in zebrafish

**Fig. 4:**
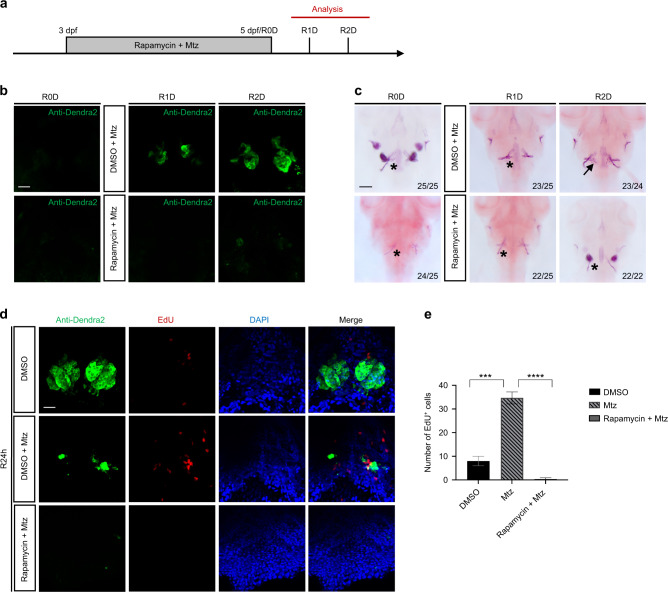
Rapamycin inhibited the repair of severely injured tooth germs. **a** Embryos at 3 dpf were treated with Rapamycin and Mtz for 48 h, and then the embryos were analyzed from R0D to R2D. **b** Antibody staining was performed on R0–2D embryos after treatment. Green fluorescence indicated tooth germs. **c** Alizarin Red staining was performed on R1–2D embryos after treatment. The black arrow points to the mineralized pharyngeal teeth, and the black “*” represents no obvious mineralization. **d** EdU staining showed the cell proliferation (red) in the tooth germ (green) at R24h. **e** Statistical analysis of EdU^+^ cells (*n* = 3). Scale bar for **c** = 100 μm; scale bar for **b** and **d** = 20 μm (***P ＜ 0.001; ****P ＜ 0.000 1)

Rapamycin is a specific inhibitor of the mTORC1 signaling. We observed repair of embryos from 0 to 2 day (R0–2D) after DMSO + Mtz or Rapamycin + Mtz treatment (Fig. 4a). Antibody staining showed that the tooth germ (green fluorescence) disappeared after DMSO + Mtz and Rapamycin + Mtz treatment, whereas fluorescence reappeared in the DMSO + Mtz group but not in the Rapamycin + Mtz group in both R1D and R2D (Fig. 4b). Similar to antibody staining results, in situ hybridization results also showed that *scpp5* and *fth1b* were expressed again in the DMSO + Mtz group in both R1D and R2D but not in the Rapamycin + Mtz group (Fig. S5a, b). In addition, Alizarin Red staining showed that in the DMSO + Mtz group, mineralized pharyngeal teeth were produced in the tooth germ region in R2D, with no apparent mineralization accumulation in the Rapamycin + Mtz group (Fig. 4c). These findings indicated that rapamycin could inhibit the repair of severely injured tooth germ. In the EdU staining results, compared with the DMSO + Mtz group, cell proliferation decreased significantly in the Rapamycin + Mtz group (Fig. 4d, e), suggesting that rapamycin might inhibit tooth germ repair by inhibiting cell proliferation.

### Severely injured tooth germ of *raptor* mutant could not be repaired

**Fig. 5:**
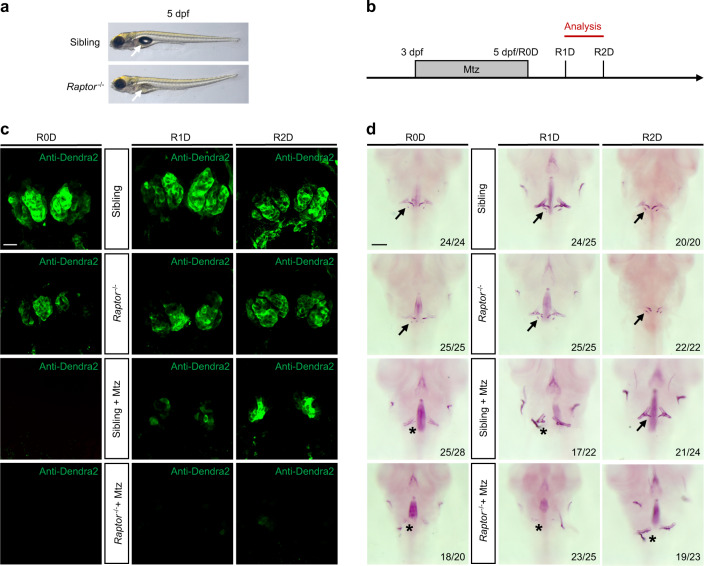
Severely injured tooth germ of *raptor* mutant could not be repaired. **a** The phenotype of wild-type fish and *raptor*^−*/*−^ embryos on day 5 after fertilization. The white arrow points to the swim bladder. **b** Mtz was used from 3 to 5 dpf. Embryos were analyzed from R0D to R2D. **c** Antibody staining was performed on R0–2D embryos. Green fluorescence shows the tooth germ. **d** Alizarin Red staining was performed on R0–2D embryos. The black arrow points to the mineralized pharyngeal teeth, and the black “*” represents no apparent mineralization. Scale bar for **d** = 100 μm; scale bar for **c** = 20 μm

**Fig. 6:**
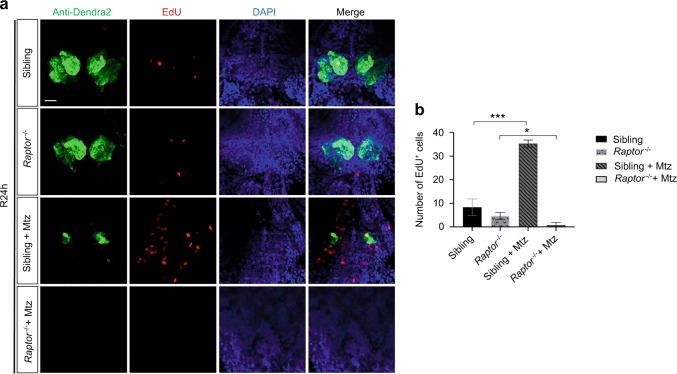
Cell proliferation in severely injured tooth germ of *raptor* mutant. **a** EdU staining showed the cell proliferation (red) of tooth germ (green) at R24h. **b** Statistical analysis of EdU^+^ cell (*n* = 3). Scale bar for **a** = 20 μm (*P ＜ 0.05; ***P ＜ 0.001)

To further validate the regulatory role of mTORC1 signaling in tooth germ restoration, we additionally applied the mTORC1 mutant (*raptor*^−*/*−^). The phenotype of *raptor*^−*/*−^ did not develop a swim bladder (Fig. 5a). Mutants with transgenic background were treated with Mtz as before (Fig. 5b). Antibody staining results indicated that the green fluorescence of tooth germ of *raptor*^−*/*−^ was slightly weaker than that of the sibling group. Mtz treatment led to disappearance of the green fluorescence. At R1–2D, green fluorescence reappeared in the sibling + Mtz group, with no apparent green fluorescence in the *raptor*^−*/*−^ + Mtz group (Fig. 5c). The results of in situ hybridization were similar to those of antibody staining. The expression of *scpp5* and *fth1b* disappeared after Mtz treatment. At R1–2D, the binding sites of *scpp5* and *fth1b* increased significantly in the sibling + Mtz group, with no apparent expression in the *raptor*^−*/*−^ + Mtz group (Fig. S5c, d). In Alizarin Red staining results, we found that the degree of mineralization is lower in the *raptor*^−*/*−^ group than in the sibling group. After Mtz treatment, the repaired tooth germs in the sibling + Mtz group could be mineralized into pharyngeal teeth. However, no significant mineralization was observed in the *raptor*^−*/*−^ + Mtz group (Fig. 5d). EdU staining and statistical analysis showed no significant difference in cell proliferation between the *raptor*^−*/*−^ and sibling groups at R24h without Mtz treatment. However, cell proliferation in the sibling + Mtz group increased significantly after Mtz treatment, with a significant decrease in the *raptor*^−*/*−^ + Mtz group (Fig. 6a, b). All those demonstrated that the knockout of the mTORC1 signaling pathway inhibited cell proliferation significantly during tooth germ repair, suggesting that mTORC1 may participate in tooth germ repair by regulating cell proliferation.

### Damaged incisor repair in mouse is controlled by mTORC1 signaling

**Fig. 7:**
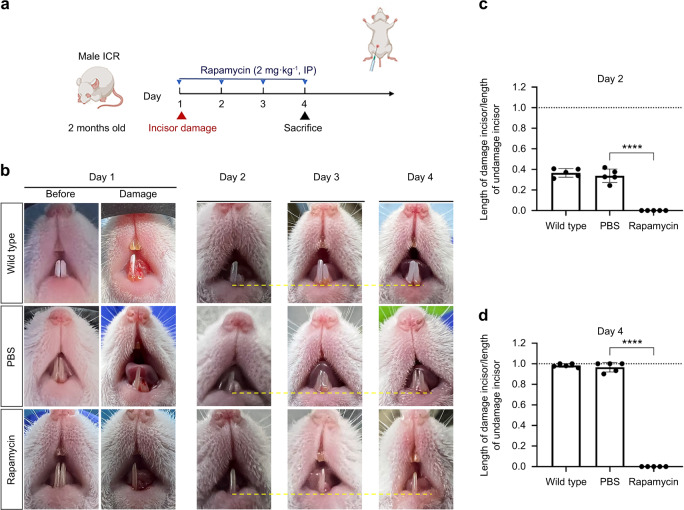
Inhibition of mTORC1 signaling could inhibit damaged incisor repair in mouse. **a** 2-month-old mice were injected intraperitoneally with rapamycin after incisor damaged, and the restoration of incisor was observed. **b** The repair process of mouse incisor after damage. The yellow dashed lines are parallel lines. **c**, **d** Statistical analysis of length of damaged incisor/undamaged incisor at day 2 and 4 (*n* = 5). The dotted line represents the incisor completed the restoration (****P ＜ 0.000 1)

**Fig. 8:**
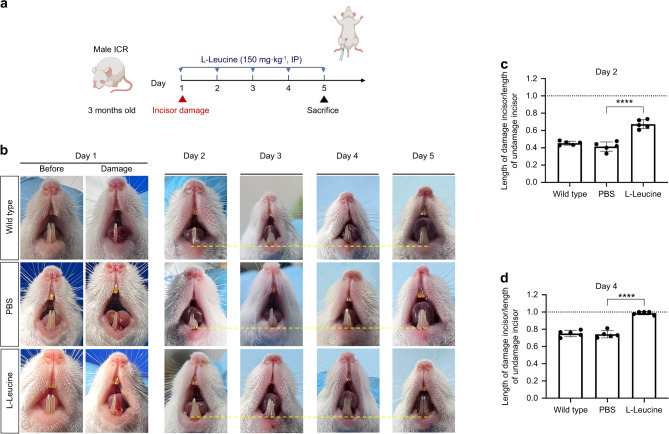
Promotion of mTORC1 signaling could promote damaged incisor repair in mouse. **a** 3-month-old mice were injected intraperitoneally with L-Leucine after incisor damaged, and the restoration of incisor was observed. **b** The repair process of mouse incisor after damage. The yellow dashed lines are parallel lines. **c**, **d** Statistical analysis of length of damaged incisor/undamaged incisor at day 2 and 4 (*n* = 5). The dotted line represents the incisor completed the restoration (****P ＜ 0.000 1)

Rapamycin was injected intraperitoneally into 2-month-old mice after incisors injury, and then the repair of incisors was observed (Fig. 7a). We found that the repairs in the phosphate-buffered saline (PBS) injection group were similar to those in the wild-type (no injection) group. By the fourth day the repair of incisor had been completed (Fig. 7b). However, in the rapamycin injection group, the damaged incisors were not repaired (Fig. 7c, d). Moreover, 3-month-old mice were intraperitoneally injected with L-Leucine after incisors damaged, and then the repair of incisors was observed (Fig. 8a). During day 2–4, compared with the control group and the intraperitoneal injection of PBS group, the length of repaired incisors in the intraperitoneal injection of L-Leucine group was longer (Fig. 8b). By day 4, the repair of damaged incisors in the L-Leucine injection group had been completed, which was 1 day faster than that in the PBS group (Fig. 8c, d), indicating that injection of L-Leucine can promote the repair of mouse incisor effectively.

## Discussion

A thorough understanding of the effector molecular axis of the key signaling pathways in tooth germ repair is the basis of the research on the treatment of tooth germ injury. In this study, with regard to the characteristics of repair after complete ablation of tooth germ, we used zebrafish as a model animal and constructed a tooth germ repair model for the first time. Using this model, we explored the regulatory role of mTORC1 signaling pathway in tooth germ repair. As far as we know, this study fills a gap in the research on the repair mechanism of injured tooth germs.

The transgenic line *Tg(dlx2b:Dendra2-NTR)* was constructed successfully at first. Mtz treatment resulted in complete ablation of the tooth germ cells, but the severely injured tooth germ would repair itself after the treatment was terminated. In the Mtz group, the first pair of repaired tooth germs could be observed at R1-3D. Another pair of tooth germs were observed at R4-5D, indicating the sequential nature of the tooth germ repair process. Gibert^40^ proposed that the first pair of tooth germs appeared as the necessary and sufficient condition for the initiation of zebrafish pharyngeal tooth development. Our results are consistent with it. In our study, the order of tooth germ repair was a pair of tooth germs appeared first, and then the other pairs followed subsequently. The order of tooth germ repair is highly consistent with that of tooth germ development in zebrafish.^20,41,42^ Alizarin Red staining showed that the repaired tooth germ could continue mineralizing into pharyngeal teeth, suggesting the newly repaired tooth germ had the same mineralization function as the uninjured tooth germ.

Zebrafish was previously used as the model animal to study tooth development and replacement. For example, Pasco-Viel et al. explored the influence of retinoic acid on pharyngeal tooth development;^21^ Zhang et al. studied the regulatory mechanism of clcn7 in tooth development of zebrafish;^22^ and Huysseune et al. explained dental epithelial stem cells of zebrafish play a important role in the replacement of pharyngeal tooth.^20^ This study firstly used zebrafish to construct a model of tooth germs repair after severe injury. The model had the following advantages: First, this model is straightforward, efficient, and easily reproducible. Second, the model of severe tooth germ injury has the characteristics of tooth germ injury specificity, with adjustable injury time. Researchers can choose any time for the treatment with Mtz and ablate the tooth germ cells specifically at the same time, providing the opportunity to study the tooth germ injury at different time intervals. Third, we can use this injury repair model to explore many problems in the future. For example, how tooth germ cells receive and recognize injury signals of tooth germ and initiate repair procedures, what cells undergo dedifferentiation during repair, and what genes, factors, and signal pathways are involved in tooth germ regeneration from the beginning to mineralization. The new model in this study has great and far-reaching significance for further study of tooth germ regeneration.

mTORC1 signaling plays a crucial role in tissue regeneration and cell proliferation.^43–45^ However, it is still unknown whether mTORC1 signaling pathway could regulate the repair of tooth germ injury. This study firstly showed that severely injured tooth germs could not be repaired after inhibition of mTORC1 signaling, and inhibition of mTORC1 suppressed cell proliferation, suggesting mTORC1 might affect tooth germ repair by regulating cell proliferation. Moreover, studies have shown that mouse incisors could keep growing back after wear is mainly due to the continuous renewal and differentiation of dental epithelial cells and mesenchymal cells in the growth center of incisors,^30^ which are similar to tooth germs. Although the tooth germ of mice cannot be repaired after injury, the incisors of mice can continue to grow. In this study, we observed that promoting mTORC1 signaling could accelerate the repair of mouse incisors after damage, indicating that mTORC1 signaling pathway is conserved in mammalian dental-related restoration. However, the specific regulatory mechanisms of mTORC1 on the repair of damaged mouse incisor are still unclear. Is the regulatory mechanism similar to that in the repair of injured tooth germ in zebrafish? Is it also involved in the repair of mouse incisor by regulating cell proliferation? In future studies, we will continue to take advantage of zebrafish in mechanistic studies to further explore the underlying molecular axis of mTORC1 in tooth germ repair. Then, the mouse and zebrafish homologous genes were obtained to investigate the functional conservation of this molecular axis during mouse incisor repair.

Recent studies on mTORC1 in tooth germs have been limited to tooth development.^46^ For instance, studies have shown that inhibition of mTORC1 causes proliferation defects and excessive cell death, resulting in facial growth defects and wrinkles.^27^ What’s more, mTORC1 inhibition disrupts enamel cell adhesion, proliferation, and differentiation, leading to defective tooth formation and malformations.^28^ In addition, inhibition of mTORC1 significantly decreases the deposition of the mineralized matrix in human deciduous tooth stem cells.^29^ However, based on the constructed model of zebrafish in which tooth germs can be repaired after severe injury, this study clarifies that mTORC1 plays an important role in tooth germ restoration. The mTORC1 signaling also acts a crucial role in the repair of mouse incisor. The study will expand the understanding of important regulatory mechanisms in tooth germ regeneration. Furthermore, it also provides theoretical and an experimental basis for the further exploration of the specific cellular and molecular mechanism of the mTORC1 in the repair process after tooth germ injury. Studies have shown that mTORC1 regulates cell proliferation by 4E-BPs.^47^ In this study, we found increased expression of p-4E-BP1 and corresponding elevated cell proliferation in the early stage of tooth germ repair, suggesting that cell proliferation is mainly regulated by mTORC1/4E-BP1 signaling during the repair. However, it still remains unclear what the direct downstream target proteins of mTORC1/4E-BP1 signaling are. Some studies have demonstrated mTORC1 promotes mRNAs recruitment to ribosome and then initiates translation by regulating the eukaryotic translation initiation factor 4F (eIF4F) complex. Mechanistically, this step mainly occurs through phosphorylation of 4E-BP. Once 4E-BP1 is phosphorylated at multiple sites by mTORC1, this will cause dissociation of 4E-BP1 from eIF4E, which in turn will lead to increased eIF4E activity. Free eIF4E is able to bind to eIF4G and eIF4A, resulting in increased RNA translation and correspondingly enhanced protein synthesis, which promotes cell proliferation.^48–50^ In a word, mTORC1 might activate mRNA translation and protein synthesis through 4E-BP1. Ribosomes are composed of ribosomal proteins and rRNA, which are necessarily involved in RNA translation. Ribosomal proteins, as the main components of ribosomes, play a crucial role in protein biosynthesis. Ribosome biogenesis 1 homolog (Urb1) has been found to regulate protein synthesis by acting downstream of mTORC1 signaling.^51^ There are dozens of ribosomal proteins, which are widely distributed in various eukaryotic cells and tissues.^52,53^ Therefore, does the mTORC1/4E-BP1 pathway promote cell proliferation by regulating the expression of downstream ribosomal proteins during tooth germ repair? If so, which ribosomal proteins play a crucial role? In future, we will further study the main downstream effector ribosomal proteins of mTORC1/4E-BP1 signaling during tooth germ repair, in order to provide potential targets for promoting the repair of injured tooth germs in clinical practice.

In conclusion, we have construct, for the first time, a repair model after tooth germ severe injury in vivo. This model can ablate the tooth germ specifically within a flexible time period, which makes it easy to study the repair of tooth germ after injury. Hence, the model has promising application prospects in future research of tooth regeneration and repairability. By this model, we found inhibition of mTORC1 signaling pathway could suppress tooth germ repair. The mTORC1 signaling is involved in the repair of injured tooth germs by regulating cell proliferation. Therefore, it is necessary to further explore the downstream effector molecular axis of mTOCR1 signaling in tooth germ repair. Designing small molecules related to mTORC1 signaling pathway to intervene in this pathway is promising as a strategy for the treatment of tooth germ injury in the future.

## Materials and methods

### Animals

A 4 136-bp fragment upstream of the zebrafish *dlx2b* translational start site was cloned from genomic DNA. Then the fragment was cloned into a vector restriction site in front of Dendra2-NTR. The vector plasmid has previously been reported.^35^ The recombinant plasmid was microinjected into embryos to construct transgenic lines. Then, 0.15 ng of purified plasmid *dlx2b:Dendra2-NTR* and I-SceI meganuclease were co-injected into embryos to allow Dendra2 expression in the tooth germ. *raptor* mutant came from the literature that has been reported.^54^

Two or 3-month-old male ICR mice were selected for the experiment. Experimental mice and zebrafish were raised and maintained in accordance with the protocols of the Institutional Animal Care and Use Committee.

### Treatment with Mtz, rapamycin, and L-Leucine

Mtz (Sigma) was dissolved in egg water and 0.2% DMSO (Sangon Biotech), and its final concentration was 12-mmol·L^−1^. The embryos at 3 dpf were treated with Mtz solution in a dark environment at 28.5 °C for 48 h. Then, washed several times and placed back in the 28.5 °C-incubator. The control group was incubated with 0.2% DMSO from 3 dpf to 5 dpf and washed with fresh egg water several times.

Rapamycin (Selleck) was dissolved in Mtz solution, and its final concentration was 2-μmol·L^−1^. This concentration was used for the soaking treatment of the larvae. Mice were intraperitoneally injected once a day with rapamycin at a final concentration of 0.2 mg·mL^−1^ and an injection dose of 10 uL·g^−1^. L-Leucine (Sigma) was dissolved in PBS and injected intraperitoneally into mice (150 mg·kg^−1^) once a day.

### In situ hybridization

The larvae were collected at certain stages and fixed with 4% paraformaldehyde (PFA) overnight. After removing PFA, the larvae were washed several times. The prepared probes were hybridized overnight in a 68.5 °C constant-temperature water bath. Table 1 presents the primers of the probes. SSCT and MABT solutions were used for gradient washing after removing the probe. Then the antibody (Anti Dig-AP (Roche)) was incubated overnight on a shaker at 4 °C. After antibody remove, wash several times with MABT. The embryos were stained with BCIP dissolved in NTMT in a 37 °C incubator under dark conditions. After completion of staining, a stop solution (containing 0.05-μmol·L^−1^ phosphate buffer, 1-mmol·L^−1^ ethylene diamine tetraacetic acid (EDTA), and 0.1% Tween-20) was used for termination. Stained larvae were observed under a microscope (Leiss, SteREO DiscoveryV20).

**Table 1:** Specific primer sequences used for in situ hybridization

Primers	Sequences
*dlx2b*	Forward (5′–3′): ATGACCGCTGTTCTGGATAG
	Reverse (5′–3′): CTACAACAGGTGTATTCGGC
*scpp5*	Forward (5′–3′): GTGCACTGTTTGCTGATTC
	Reverse (5′–3′): TGCATATGGCTGACTGATGG
*fth1b*	Forward (5′–3′): GTGTGTCAGTCTGCAGTTGT
	Reverse (5′–3′): TGCATATGGCTGACTGATGG

Genes associated with tooth germ of zebrafish were detected as follows: *dlx2b* (distal-less homeobox 2b); *scpp5* (secretory calcium-binding phosphoprotein 5); *fth1b* (ferritin, heavy polypeptide 1b)

### Antibody staining

The embryos fixed by 4%FPA were washed with PBST several times. Then Dumont Tweezers #55 (World Precision Instruments, Sarasota, USA) were used to carefully remove the yolk and heart, followed by soaking in acetone overnight at −30 °C. After several times of PT washing, the primary antibody (Dendra2 (1:1 000, TA180094, Origene), Phospho-4E-BP1 (1:1 000, 2855S, Cell Signaling Technology), was incubated overnight at 4 °C. After several times of PT washing, the second antibody (Alexa Fluor 488 (1:1 000, A-21202, Invitrogen), Alexa Fluor 568 (1:1 000, A10042, Invitrogen)), was incubated overnight in the dark at 4 °C. The stained larvae were observed and imaged using an LSM880 confocal microscope (Carl Zeiss).

### FISH (fluorescence in situ hybridization)-antibody staining

The embryos were treated with 3% H_2_O_2_ in methanol for at least 1 h, followed by washing with 25%, 50%, and 75% PBST in methanol at RT for 5 min. Tweezers were used to carefully remove the yolk and heart in 4% PFA solution. The samples were washed with PBST several times. The *dlx2b* gene probe was hybridized overnight in a 65 °C constant-temperature water bath, and the probe’s sequence is shown in Table 1. The Anti Dig-POD (Roche) was incubated at 4 °C overnight, then used PT to wash several times and incubation with Cy3 (Roche) at room temperature overnight. The following steps were the same as those for antibody staining.

### TUNEL staining

Embryos were fixed by 4%FPA after 12 h Mtz treatment. The next steps were similar to those for antibody staining. The embryos were stained according to the instructions of TUNEL kit (Roche). Then the primary and the second antibodies were incubated according to the antibody staining steps. Finally, the embryos were photographed using a confocal laser.

### Alizarin Red staining

Embryo treatment was the same as described previously for antibody staining. After the yolk and heart were carefully removed, 50% ethanol was added to the larvae under RT for 10 min. After removing 50% ethanol, the larvae was soaked in Alizarin Red stain solution (Sangon Biotech) in a dark environment. The samples were shaken slowly overnight under RT. Then, a bleach solution was added (a final concentration of 1.5% H_2_O_2_ and 1% KOH) to remove pigmentation with tubes open at RT for 20 min. Finally, a clearing solution consisting of 20% glycerol and 0.25% KOH was added and shaken at RT overnight. Then took pictures under a microscope (Leiss, SteREO DiscoveryV20).

### Quantitative real-time polymerase chain reaction (RT-PCR)

The embryos were excised from the lower margin of the otolith to the upper margin of the liver with a scalpel blade. Tissues were treated with Trizol (Takara) to extract total RNA, and total RNA was then reverse-transcribed into cDNA. SYBR reagent was used for the real-time quantitative PCR reaction. The primer sequences are shown in Table 2. Three separate experiments were conducted using β-actin as the housekeeping gene.

**Table 2:** Specific primer sequences used for real-time PCR

Primers	Sequences
Notch1	Forward (5′–3′): TGTGGATGCTGCTGTCGT
	Reverse (5′–3′): GTCCTTCCCGCTGTCTTT
β-catenin	Forward (5′–3′): ATCATGCGCTCCCCACAGATGGTA
	Reverse (5′–3′): GCCTCCGCTGGCCAGAATGATAAG
PI3KR1	Forward (5′–3′): ACATGGCTCTGCAAGATGCT
	Reverse (5′–3′): GGAGGCATCTCGGACCAAAA
PI3KCA	Forward (5′–3′): CGCAATGAGAGGATGAGCGA
	Reverse (5′–3′): ACGCTGTCACGATGGAACAA
mTOR	Forward (5′–3′): AGATCATCAACCGAGTGCGG
	Reverse (5′–3′): AGGGCACCATCCAATGTAGC
Akt1	Forward (5′–3′): TCGGCAGGTGTCTTCTCAAT
	Reverse (5′–3′): ACCCATTGCCATACCACGAG
MAPK3	Forward (5′–3′): GAGTCGGTGAAGGGACAAAA
	Reverse (5′–3′): TGATCCCGATGATGTTCTCA
MAPK8	Forward (5′–3′): CTGCTGCAGATGACCATCCTTT
	Reverse (5′–3′): ACAGAGCATATTTGAGGGGGCT
MAPK14a	Forward (5′–3′): CCCGTGCAGTATCAGAACTT
	Reverse (5′–3′): CAGACTTGTGGCAGGTGTAA
β-actin	Forward (5′–3′): CCACCTTAAATGGCCTAGCA
	Reverse (5′–3′): CATTGTGAGGAGGGCAAAGT

Genes associated with signaling pathways were detected. Values were normalized to β-actin

### EdU staining

EdU is a thymine nucleoside analog. EdU conjugate reaction with dye can effectively detect the proliferating cell.^55^ Therefore, it can be used for efficient and rapid detection and analysis of cell proliferation. Embryo treatment was carried out in the same way described earlier. After removing Mtz, the embryos were incubated with EdU A in 0.2% DMSO for 1 h (R24–25h) at 28.5 °C in the dark. Tweezers were used to carefully remove the yolk and heart, followed by soaking in acetone overnight at −30 °C. After several times of PT washing, cell proliferation was detected using an EdU Alexa Fluor 647 Imaging kit (Invitrogen). Then antibody staining was performed. Finally, the stained larvae were observed and imaged under an LSM880 confocal microscope (Carl Zeiss).

### Statistical analysis

The results were statistically analyzed using GraphPad Prism 9 software. The Student’s *t* test was used to compare two independent samples. *P* < 0.05 represented a statistical significance.

## Supplementary information

Supplementary materials

## Data Availability

All data are available in the main text.
