# Supplementary material for: mTORC1 signaling pathway regulates tooth repair
Source: Int J Oral Sci. 2023 Mar 16;15:14. doi: 10.1038/s41368-023-00218-3 (PMC10020452; doi:10.1038/s41368-023-00218-3)
Supplement: Supplementary file 1 — Supplementary materials [file 41368_2023_218_MOESM1_ESM.docx]

**Supplemental Information**

**mTORC1 Signaling Pathway Regulates Tooth Repair**

**Honghong Liu^1,2,3,4^, Yu Yue^1,2,3,4^, Zhiyun Xu^1,2,3,4^, Li Guo^1,2,3,4^, Chuan Wu^5^, Da Zhang^5^, Lingfei Luo^5^, Wenming Huang^1,2,3,4^, Hong Chen^1,2,3,4^, Deqin Yang^1,2,3,4^***

1 Department of Endodontics, Stomatological Hospital of Chongqing Medical University, Chongqing, China

2 Stomatological Hospital of Chongqing Medical University, Chongqing, China

3 Chongqing Key Laboratory of Oral Diseases and Biomedical Sciences, Chongqing, China

4 Chongqing Municipal Key Laboratory of Oral Biomedical Engineering of Higher Education, Chongqing, China

5 Institute of Developmental Biology and Regenerative Medicine, Southwest University, Chongqing, China

***Corresponding author:** Prof. Deqin Yang, Stomatological Hospital of Chongqing Medical University, North Songshi Road 426, Chongqing 401147, Chongqing, People's Republic of China. Email: yangdeqin@hospital.cqmu.edu.cn.

**These authors contributed equally: Honghong Liu, Yu Yue.**


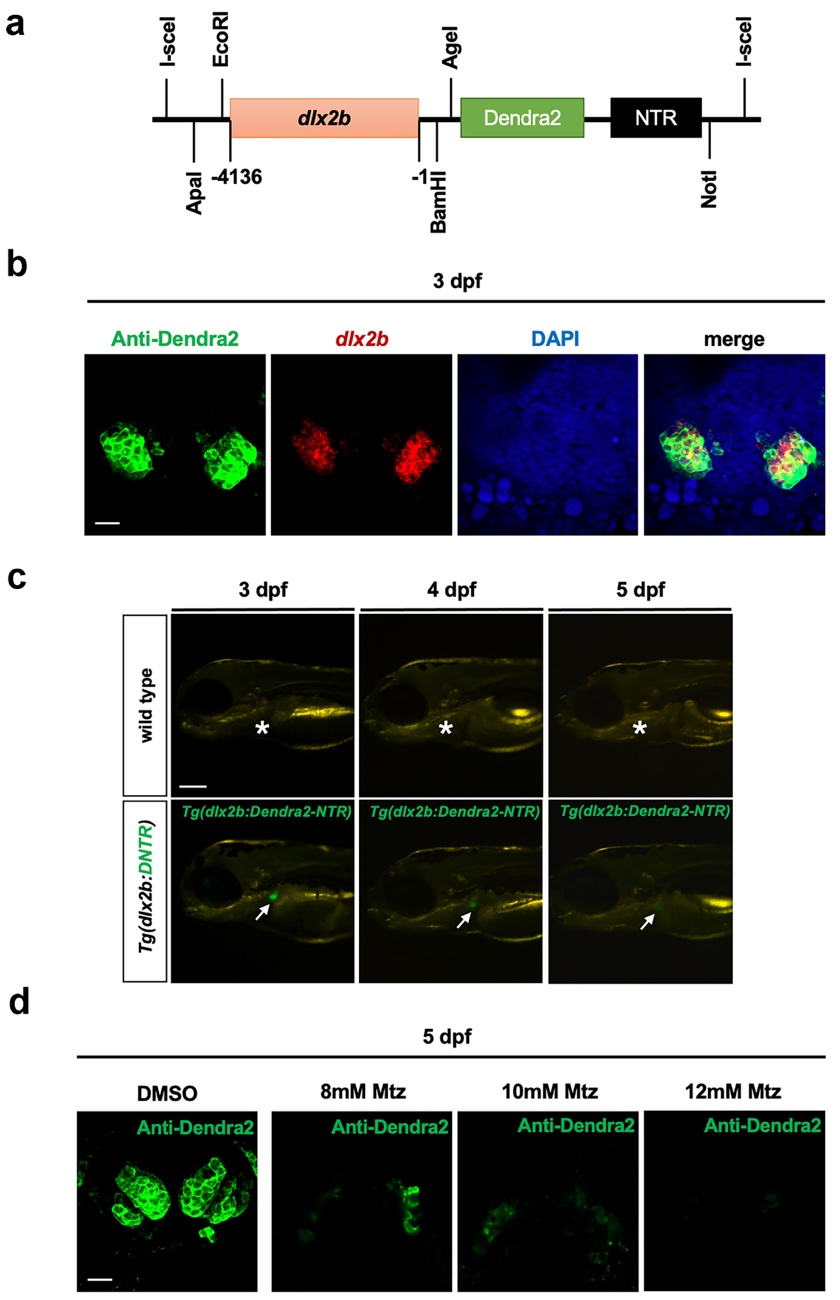


**Fig. S1.** **Construction of the severe tooth germ injury model based on transgenic line *Tg(dlx2b:Dendra2-NTR)*.** (a) Schematic diagram of the recombinant plasmid. (b) FISH antibody staining showed the expression positions of Dendra2 (green) and *dlx2b* (red fluorescence). (c) Ventral images of wild-type and transgenic line *Tg(dlx2b: Dendra2-NTR)* during 3-5 dpf intervals. The white arrows point to the site of green fluorescence expression, and the white “*” indicates no obvious green fluorescence expression. (d) Antibody staining of Dendra2 showed tooth germ (green) injury severity after Mtz treatment at different concentrations. Scale bar for c = 100 μm; scale bar for b and d = 20 μm


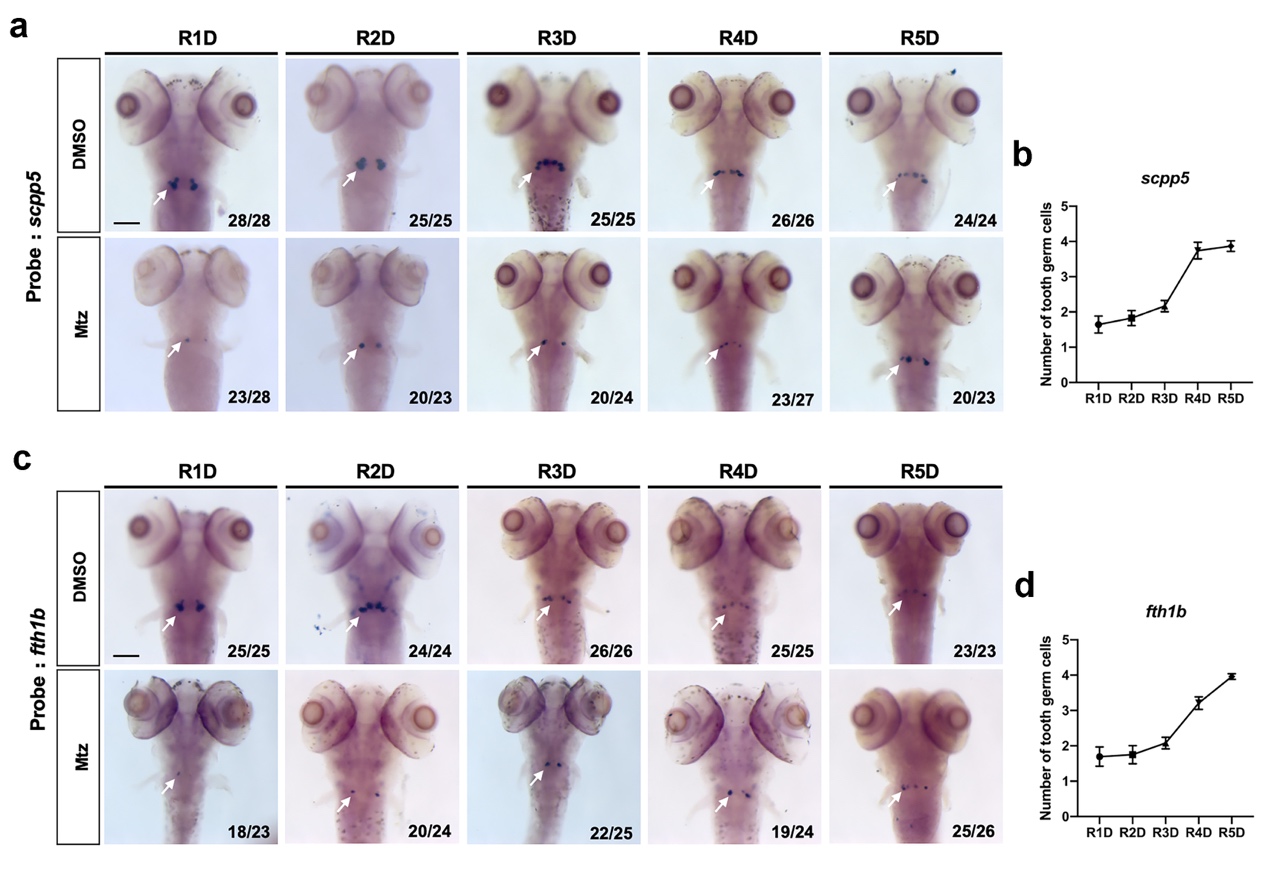


**Fig. S2. *Tg(dlx2b:Dendra2-NTR)* could be repaired effectively after severe injury of the tooth germ.** (a, c) In situ hybridization of R1-5D embryos. The probes were *scpp5* and *fth1b*, respectively. The white arrow points to the probe-binding site, and the white “*” indicates no obvious probe-binding site. The number in the lower right corner is the number of positive/total number of experiments. (b, d) The number of probe-bound tooth germs in embryos at different periods was analyzed in the Mtz-treated group, and the probes were *scpp5* and *fth1b*, respectively. Scale bar for a and c = 100 μm


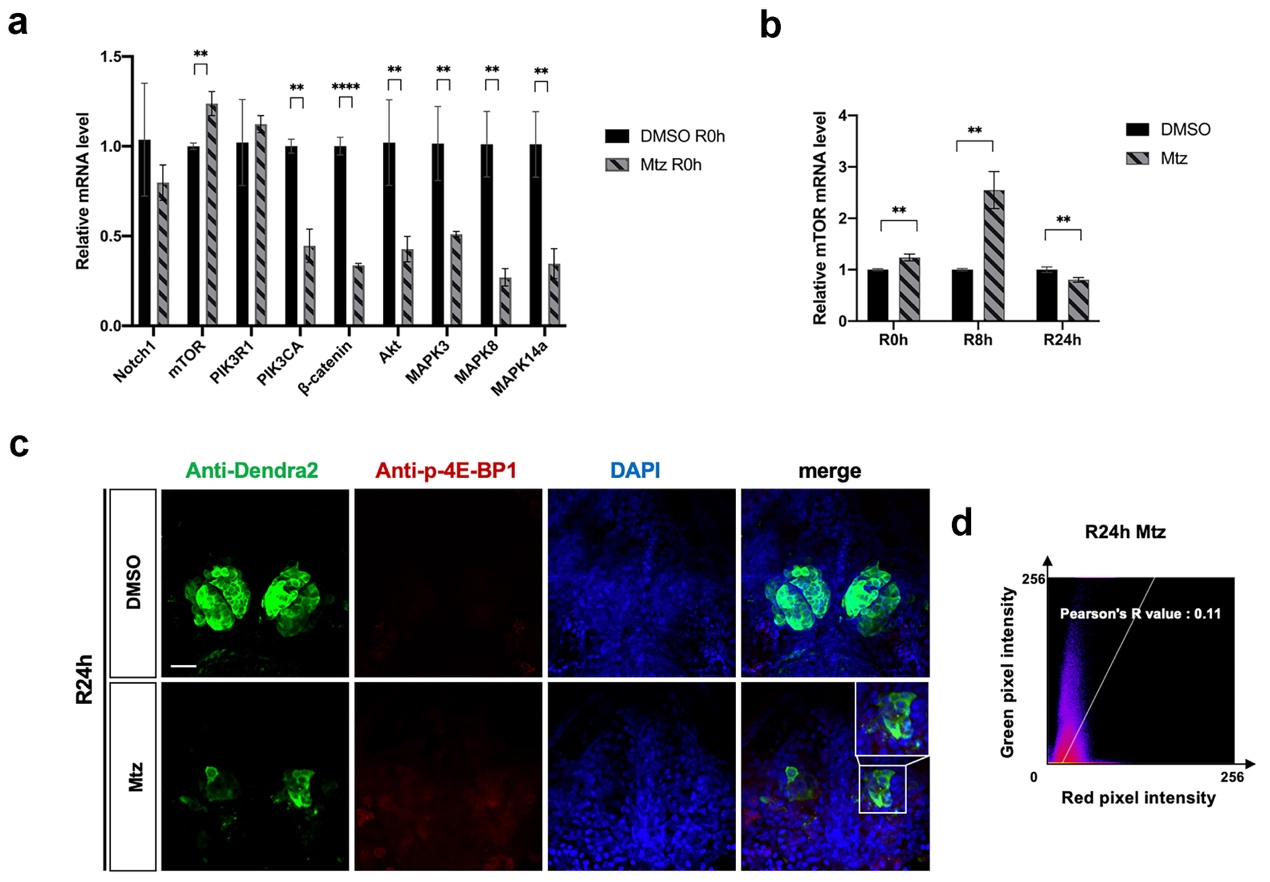


**Fig. S3. mTORC1 signaling was activated in the early stage of tooth germ repair.** (a) RT-PCR was used to detect the expression of each gene in each group at R0h. (b) RT-PCR was used to detect the expression of mTOR at R0h, R8h, and R24h. (c) Antibody staining was performed on R24h embryos, and p-4E-BP1 (red) was located by confocal laser scanning. p-4E-BP1 was not co-stained with the tooth germ (green) at R24h. (d) Co-localization analysis of p-4E-BP1 (red) and tooth germ (green) in the Mtz-treated group at R24h. Scale bar for c = 20 μm (***p*＜0.01; *****p*＜0.0001)


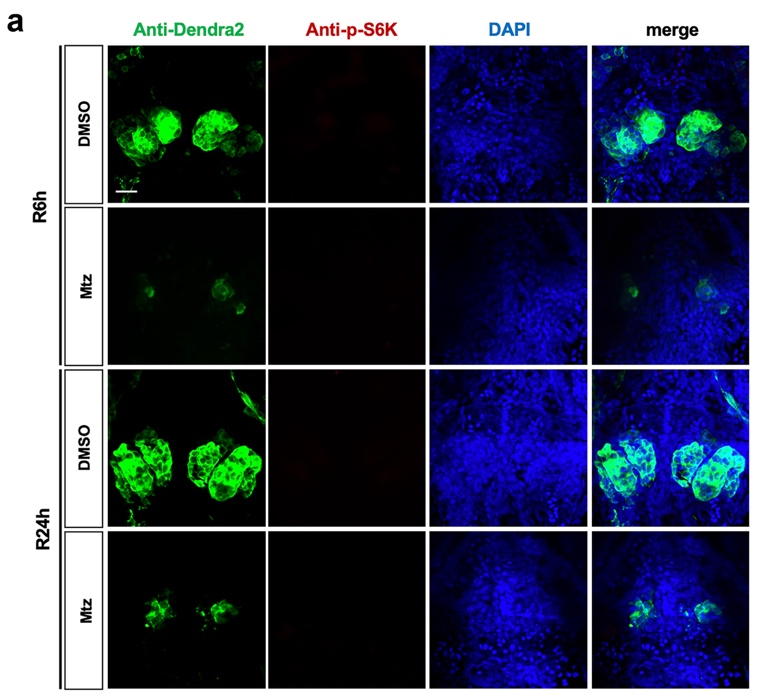


**Fig. S4. mTORC1 signaling was activated in the early stage of tooth germ repair.** (a) Antibody staining was performed on R6h and R24h embryos, and p-S6K (red) was located by confocal laser scanning. p-S6K was not expressed in the anatomical position of tooth germ (green). Scale bar for a = 20 μm


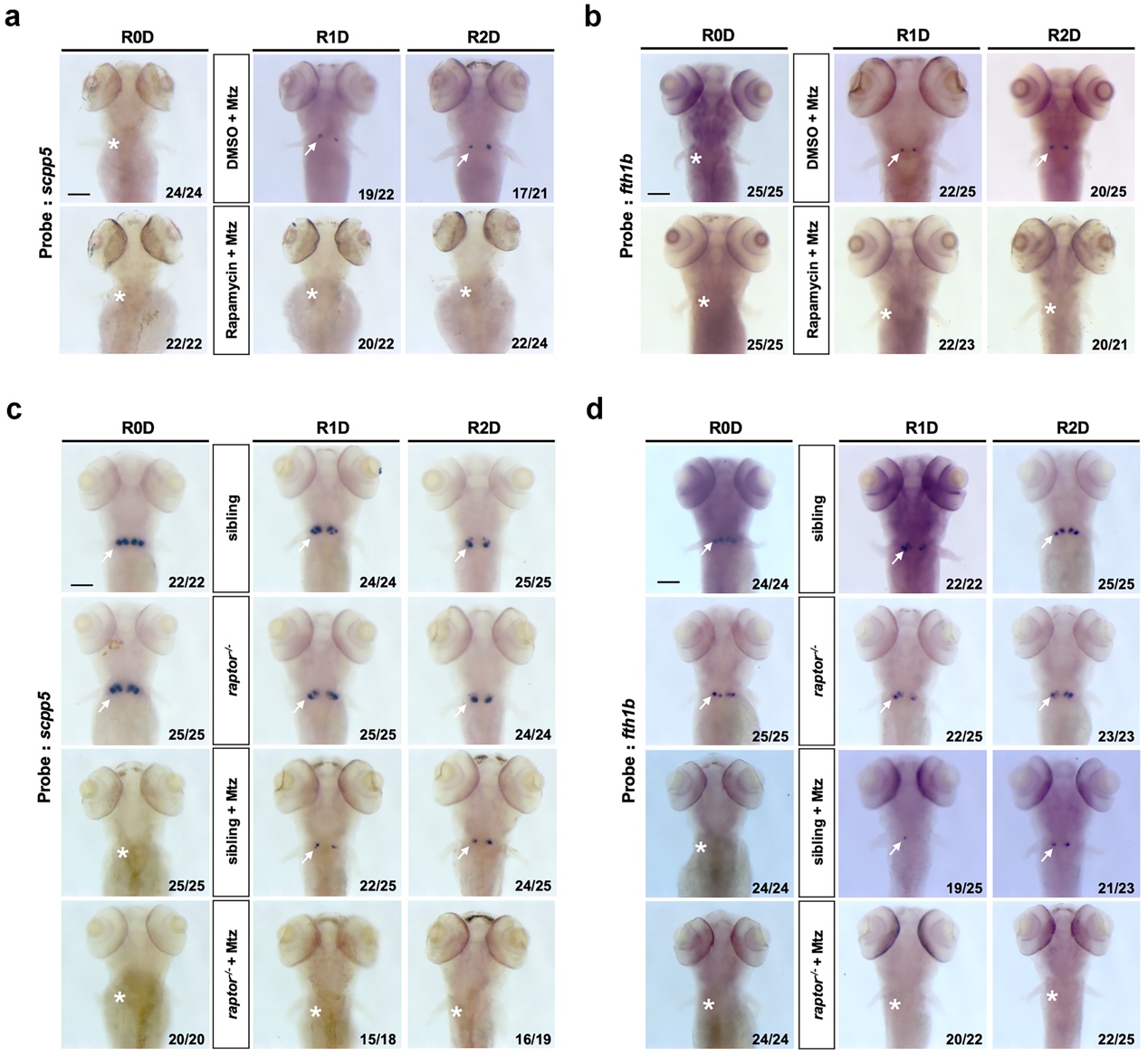


**Fig. S5. Severely injured tooth germs could not be repaired in the presence of mTORC1 signaling inhibition.** (a-b) In situ hybridization was performed on R0-2D embryos after DMSO + Mtz or Rapamycin + Mtz treatment. The probes were *scpp5* and *fth1b*, respectively. The white arrow points to the probe-binding site, and the white "*" indicates no apparent probe-binding site. (c-d) In situ hybridization was performed on R0-2D embryos after Mtz treated. The probes were *scpp5* and *fth1b*, respectively. The white arrow points to the probe-binding site, and the white "*" indicates no apparent probe-binding site. The number in the lower right corner is the number of positives/total number of experiments. Scale bar for a, b, c and d = 100 μm
